# Problems in CSF and Ophthalmic Disease Research

**DOI:** 10.3389/fopht.2022.896680

**Published:** 2022-06-28

**Authors:** Ryan Machiele, Benjamin Jay Frankfort, Hanspeter Esriel Killer, David Fleischman

**Affiliations:** ^1^ Department of Ophthalmology, University of North Carolina at Chapel Hill, Chapel Hill, NC, United States; ^2^ Department of Ophthalmology, Baylor College of Medicine, Houston, TX, United States; ^3^ Department of Ophthalmology, Kantonsspital Aarau, Aarau, Switzerland; ^4^ Center for Biomedicine University of Basel, Basel, Switzerland

**Keywords:** translaminar pressure difference, translaminar pressure gradient, cerebrospinal fluid, glaucoma, lamina cribrosa

## Abstract

There has been significant interest and progress in the understanding of cerebrospinal fluid pressure and its relationship to glaucoma and other ophthalmic diseases. However, just as every physiologic fluid pressure fluctuates, cerebrospinal fluid pressure (CSFP) is similarly dynamic. Coupling this with the difficulty in measuring the pressure, there are many obstacles in furthering this field of study. This review highlights some of the difficulties in CSFP research, including fluid compartmentalization, estimation equations, and pressure fluctuation. Keeping these limitations in mind will hopefully improve the quality and context of this burgeoning field.

## Introduction

Glaucoma is the second leading cause of blindness worldwide and is characterized by a specific pattern of nerve damage with corresponding visual field loss. Elevated intraocular pressure (IOP) is the only clinically modifiable risk factor and as such is central to diagnosis and treatment. Many theories exist to explain the pathophysiology of pressure-driven nerve damage including vascular dysfunction (1), metabolic and axonal dysregulation (2), and mechanical damage (3). In general, there is agreement that damaging IOP injures the optic nerve at the nerve head, where it disrupts axonal flow to cause retrograde retinal ganglion cell (RGC) loss.

A growing body of research supports the notion that IOP is only part of the equation in the process of pressure-driven optic nerve damage. In this paradigm, RGC damage is the product of the net imbalance between two pressurized compartments: the intraocular space and the optic nerve sheath subarachnoid space. These compartments typically have different pressures, and the force exerted by IOP at the optic nerve head is opposed by the cerebrospinal fluid pressure (CSFP), or intracranial pressure (ICP).

The difference between these two compartments is termed the translaminar pressure difference (TLPD). An increased TLPD (IOP>CSFP) is postulated to precipitate glaucomatous nerve damage, perhaps contributing to posterior bowing of the lamina cribrosa (4–6). Elevations in IOP can certainly cause this imbalance, but low ICP in the presence of a “normal” IOP has also been linked with glaucomatous nerve damage. Several studies have found a strong correlation between lower ICP and glaucoma, while numerous animal-model studies have identified a causal relationship. Ren et al. prospectively studied patients with normal tension glaucoma (NTG) and primary open angle glaucoma (POAG), and found that visual field loss was strongly associated with an increased TLPD (7). Berdahl et al. has published several large-scale retrospective reviews of patients who had undergone lumbar puncture (LP), and found that lower ICP and higher TLPG is associated with POAG and NTG (8, 9). In 1979, Yablonski et al. sought to develop a model for causality in cats by lowering ICP and unilaterally lowering IOP, and found that eyes with a greater difference between IOP and ICP (TLPD) developed more cupping and posterior bowing of the LC (10). Twenty-six years later, Zhao et al. used the electroretinogram to measure changes in retinal function when varying IOP in rats with normal ICP compared with reduced ICP, and found a larger TLPG resulted in worse retinal function (11). Most recently, Zhu et al. conducted an *in vivo* study of monkeys using optical coherence tomography to image and quantify real-time deformations of the LC, and found that lowering ICP resulted in larger amplitude bowing of the LC in response to IOP variations (12). Further evidence of a relationship of optic nerve cupping related to changes in CSF pressure and flow dynamics is highlighted by works by Gallina and colleagues that examined the development of NTG in patients with shunt-treated normal pressure hydrocephalus (13).

The relevant biomechanics of the LC include two key stress forces with significantly different vectors: the forces acting from opposing sides of the LC (IOP and CSFP) and the hoop stress within the sclera, acting circumferentially around the LC (14). Stress is defined as the force across a small boundary per unit area of that boundary (15). Stress force when applied causes strain, or measurable deformation. At the most fundamental level, deformation of the LC is thought to directly damage nerve and vascular tissue. The opposing forces of IOP and CSFP cause stress at the level of the LC that is inversely proportional to the thickness of the LC: stress decreases with increasing LC thickness. Circumferential hoop stress at the level of the LC is directly proportional to the IOP and inversely proportional to the scleral thickness (or rigidity): as IOP increases, the hoop stress translated to the LC increases, while as scleral thickness increases, LC stress is decreased. It is worth noting that of the two primary stress forces, the trans-laminar force is influenced by CSFP whereas the hoop stress is not. Baneke and colleagues point out that since hoop stress decreases with increasing scleral rigidity, the increased scleral rigidity seen in aging can be thought to make hoop stress less of a factor, and CSFP mediated stress more significant (14).

While much research has been dedicated to the role of CSFP in the pathogenesis of glaucoma and the biomechanics of the LC, the fluid pressure is very difficult to study. The purpose of this review is to highlight some of the difficulties in the study of the contribution of CSF in glaucomatous pathogenesis.

## Measurement of CSF Pressure

Central to our understanding of the translaminar pressure gradient is accurate measurement of CSF pressure. This has proven a challenge for the scientific community due to limitations in techniques for invasive pressure monitoring as well as shifting definitions of CSFP.

CSFP is classically assessed *via* the lumbar puncture (LP). The LP was first introduced by Heinrich Quincke in 1891 but the technique was not immediately adopted in clinical practice (16). In 1950, Pierre Janny shed light on the clinical applications of CSFP measurement in his examination of the relationship between ophthalmologic signs and CSFP (17). The LP remains the mainstay to the present day for clinical assessment of CSFP. This technique provides an instantaneous measurement of the intrathecal CSFP, which indirectly describes intracranial CSFP under Pascal’s principle which assumes that CSF circulates freely throughout the subarachnoid space. For much of the 20^th^ century, the intracranial CSFP was, by definition, synonymous with intrathecal CSFP as alternative methods for assessing intracranial CSFP did not exist. It should not be overlooked, however, that the LP, while used ubiquitously to assess *intracranial* CSFP, is ultimately an indirect measurement. Lundberg was the first to attempt direct and continuous measurement of ventricular CSFP in 1960 (18). Continued developments in direct measurement of ventricular and brain-tissue pressure have redefined true intracranial CSFP and spawned several studies which examine the variation between intrathecal CSFP and ICP measured by brain tissue pressure-sensing transducers in patients in intensive care units with continuous electrode monitoring (19). These studies have been performed exclusively in neurocritical patients, the only clinical population where such invasive monitoring is routinely justified. Unfortunately, the results of these studies are difficult to generalize as several other studies have demonstrated that measurement of CSFP in critical care and sedation settings may be unreliable, due to factors such as hypercarbia and direct action of anesthetic agent (20). Additionally, instrumentation in critically ill populations confers additional risks including infection, hemorrhage and development of neurological deficits, which make prospective studies nonviable. Outside of the critically ill population, Lenfeldt and colleagues performed a novel study which employed a pressure control strategy in patients with communicating hydrocephalus and found a high degree of agreement between intracranial CSFP and CSFP measured by LP (21). Again, these results are difficult to generalize given the unique physiology of the population studied.

The LP faces three key limitations as a reliable tool for studies involving CSFP: it is an invasive sterile procedure which requires a high level of skill, it is indirect in its assessment of intracranial CSFP, and it is highly temporal, providing only a snapshot of intrathecal CSFP (19). To tackle the problem of the impracticability of an invasive, sterile technique in most outpatient clinical settings, a myriad of non-invasive techniques for CSFP estimation are currently being developed. These innovations typically follow one of two paths and provide either *qualitative* markers that suggest the possibility of increased cranial CSFP or *quantitative* measurements of intracranial CSFP or estimations based on previous measurements (22). Optic nerve edema is commonly employed as a qualitative indicator of elevated CSFP. By extension, many studies have sought to quantify optic nerve changes as a surrogate measurement of CSFP, typically through measurement of optic nerve sheath diameter (ONSD) *via* ultrasound, CT or MRI. Chen et al. performed ultrasound measurement of ONSD 5 minutes before and after LP and found that ONSD correlated closely with real-time changes in CSFP (23). Weidner et al. performed ultrasonographic measurement of ONSD in awake, spontaneously breathing patients with continuous invasive ICP monitoring, finding a strong correlation between ONSD and CSFP (24). Bauerle et al. compared ultrasound and MRI derived values of ONSD, and found a high degree of agreement (25). As IOP measurement is readily obtained in the outpatient setting without a sterile field and without risk of harm to the patient, further exploration of the role TLPD plays in the pathogenesis of glaucoma will be best served by CSFP measurement techniques that are similarly non-invasive. Thus far, ultrasound imaging of the ONSD certainly shows the most promise as a potential surrogate for general estimates of CSFP status.

## The Relationship Between Intracranial and Orbital CSF Pressure

One of the key questions facing CSF and ophthalmic disease research is whether CSFP measured within the lumbar space reliably represents the CSFP within the optic nerve sheath. In most clinical studies examining the TLPG, the pressure measured during LP was used as a surrogate for the pressure posterior to the lamina cribrosa, or the orbital CSFP. Considering the distance and differences in the anatomy between the lumbar spine and the orbital subarachnoid space, it is logical to question the utility of an LP to estimate orbital CSFP. In a study on 10 patients with idiopathic normal pressure hydrocephalus, Lenfeldt et al. compared the lumbar pressure to parenchymal pressure in the brain and found an agreement between the measurements (21). The authors conclude their study with a caveat: the results depend on a communicating CSF system. Clinically, especially in the elderly population, there is good reason to doubt whether such a communicating CSF system exists (26). Studies applying CT myelography demonstrate that a variety of processes like vertebral degenerations, disc herniations, arachnoiditis and other anatomic variations or obstructions can markedly narrow this CSF pathway—and possibly completely obstruct CSF flow (27). But even if the CSF pathway from the lumbar site to the intracranial CSF spaces is patent, there is yet another restriction point. The optic canal, within the lesser wing of the Sphenoid, can be a critical bottleneck for free communication from the intracranial compartment to the lamina cribrosa. Computer-assisted imaging studies revealed that the optic canal can be particularly narrow in some patients with normal tension glaucoma when compared to nonglaucoma controls (28). Similarly, computer-assisted cisternography demonstrated impaired CSF dynamics between the intracranial CSF spaces and the orbital subarachnoid space in a series of patients with papilledema and normal tension glaucoma, which proved the existence of an optic nerve compartment syndrome (29, 30). Studies of animal models have utilized an array of subarachnoid pressure-sensitive probes placed at the level of the optic nerve to assess for variations between intracranial and intraorbital CSFP and found variability (31). Other studies have used various tracer dyes to assess for continuity of the subarachnoid space into the optic nerve and confirmed various bottlenecks (32). In general, these studies suggest a positive relationship between CSFP within the optic nerve and intracranial CSFP obtained *via* LP, but also demonstrate highly variable intraorbital CSF inflow, suspected to be due to fibrillar tissue and CSF compartmentalization in the subarachnoid space. To assume that the lumbar pressure is a reliable surrogate for the pressure posterior to the lamina cribrosa is therefore speculative, likely inaccurate in many subjects, and in need of further study by new measurement technologies. But even if the pressure on both sides of the lamina cribrosa was known accurately, the area of the lamina cribrosa – an important component in the definition of pressure, is not. Indeed, because of the space occupying trabeculae in the subarachnoid space, the area of the lamina cribrosa resembles a fractal ring anulus with a high variability between individuals (33). Another potential confounding variable is the thickness of the lamina cribrosa and the transmission and action of pressure across this tissue. The lamina is known to be thinner in patients with glaucoma, possibly increasing the transmissibility of pressures (34). Studying the role of CSFP and IOP counterbalance factoring lamina cribrosa thickness and biomechanics is very difficult, and an area in need of further study. Only William Morgan has studied the TLPG in earnest, taking into account the thickness of the lamina with the pipette-manometer measurement technique, and it was clear that the thickness of this structure was critical (35). These factors confound the accurate measurement of TLPG.

**Figure 1:**
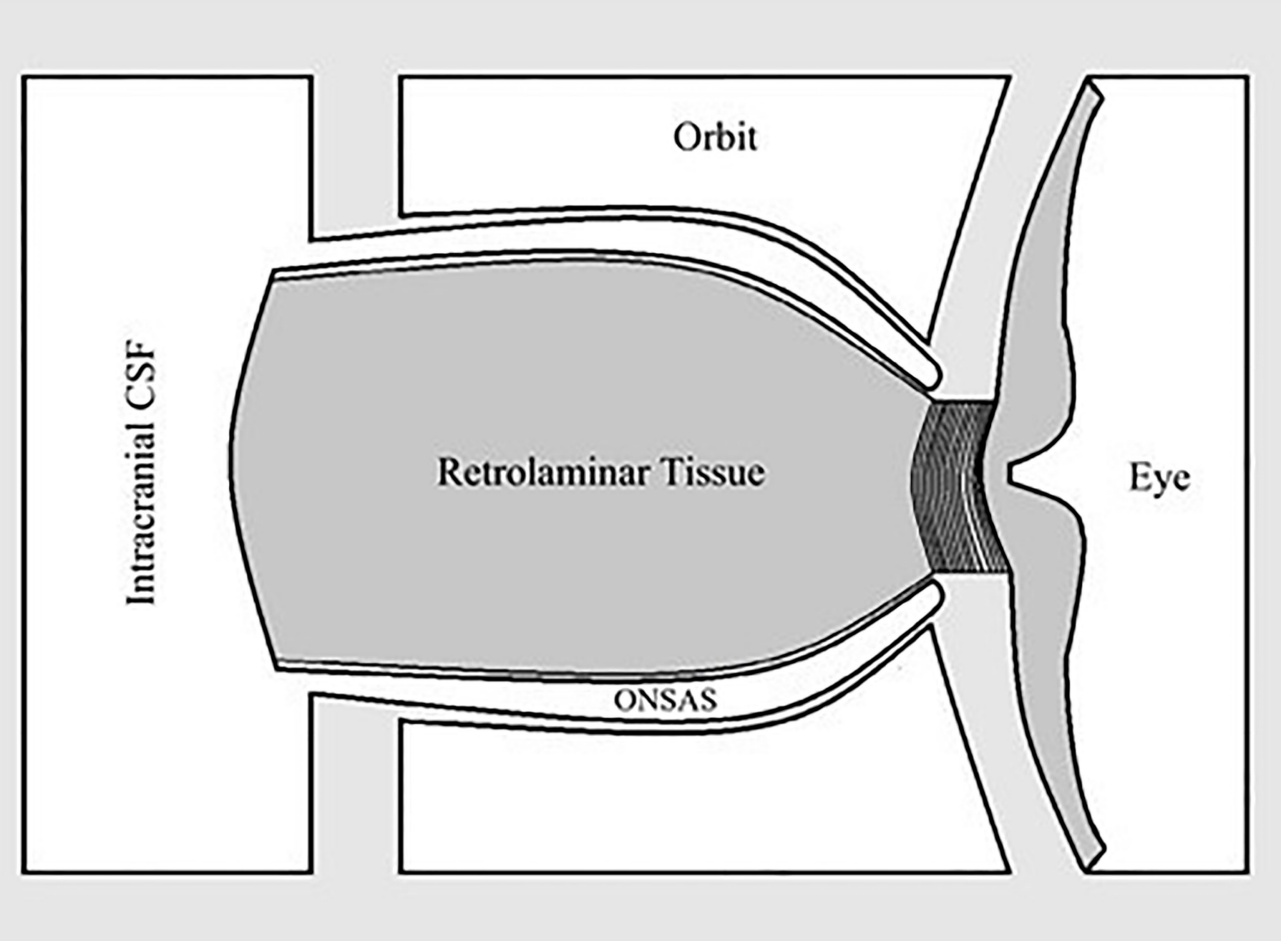
Relationship between optic nerve subarachnoid space (ONSAS) and the eye, highlighting that the pressure of interest is perhaps paralaminar instead of translaminar.

**Figure 2:**
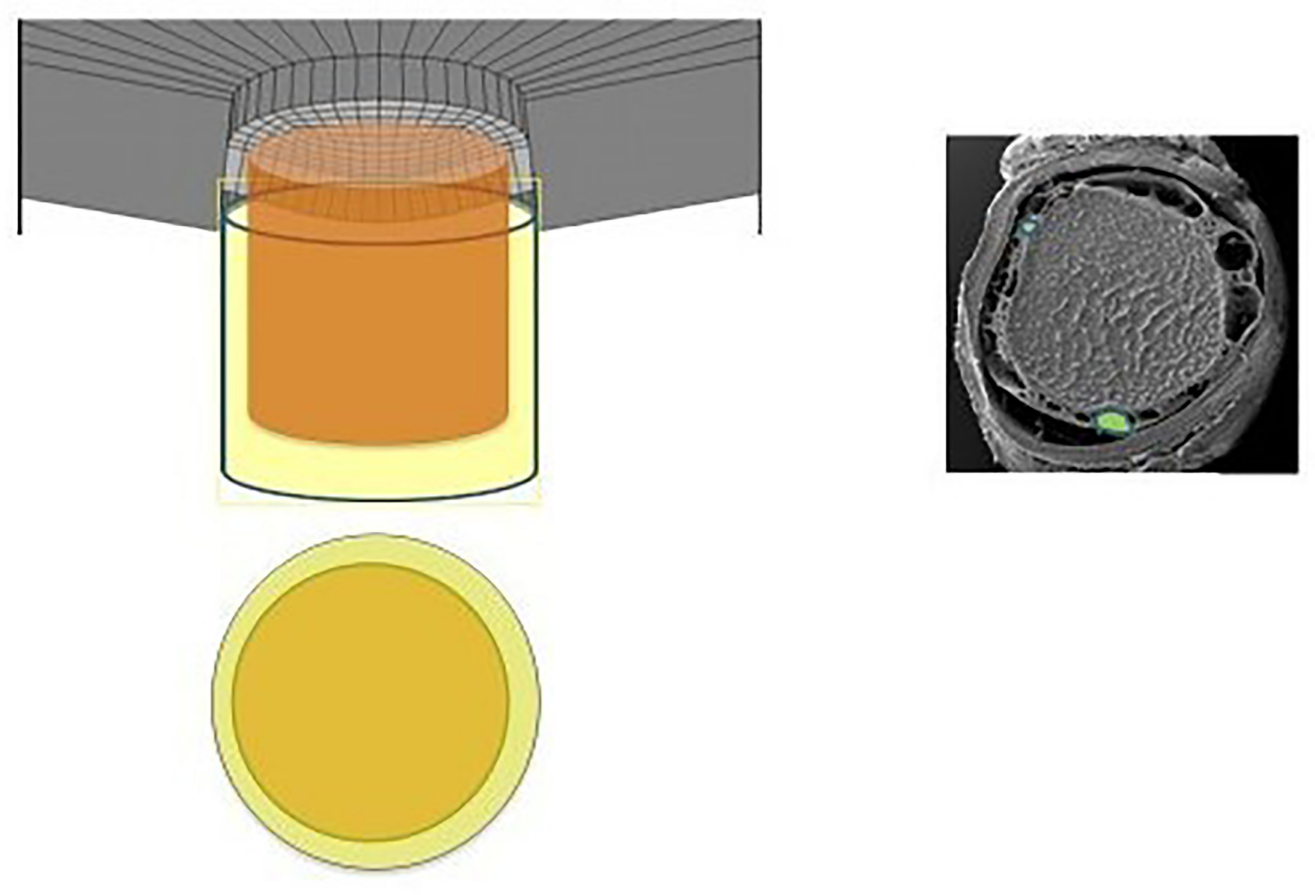
Diagram and electronic micrographic image revealing the subarachnoid space, again highlighting the CSF-containing space in relation to the intraocular space (36).

Lastly, we question the importance of the “translaminar pressure.” The translaminar pressure gradient or difference would be between the eye and the retrolaminar optic nerve tissue. If we consider the optic nerve subarachnoid space (ONSAS) and the eye, the correct terminology would be the transscleral pressure (**Figure 1**). Of course, the fluid pressure around the nerve also affects the nerve tissue pressure itself, so it is possible that any or all of these pressure relationships are most critical (**Figure 2**).

## Utility of Singular Pressure Readings: What Is Known About Diurnal, Longitudinal and Positional Changes in CSFP

**Figure 3:**
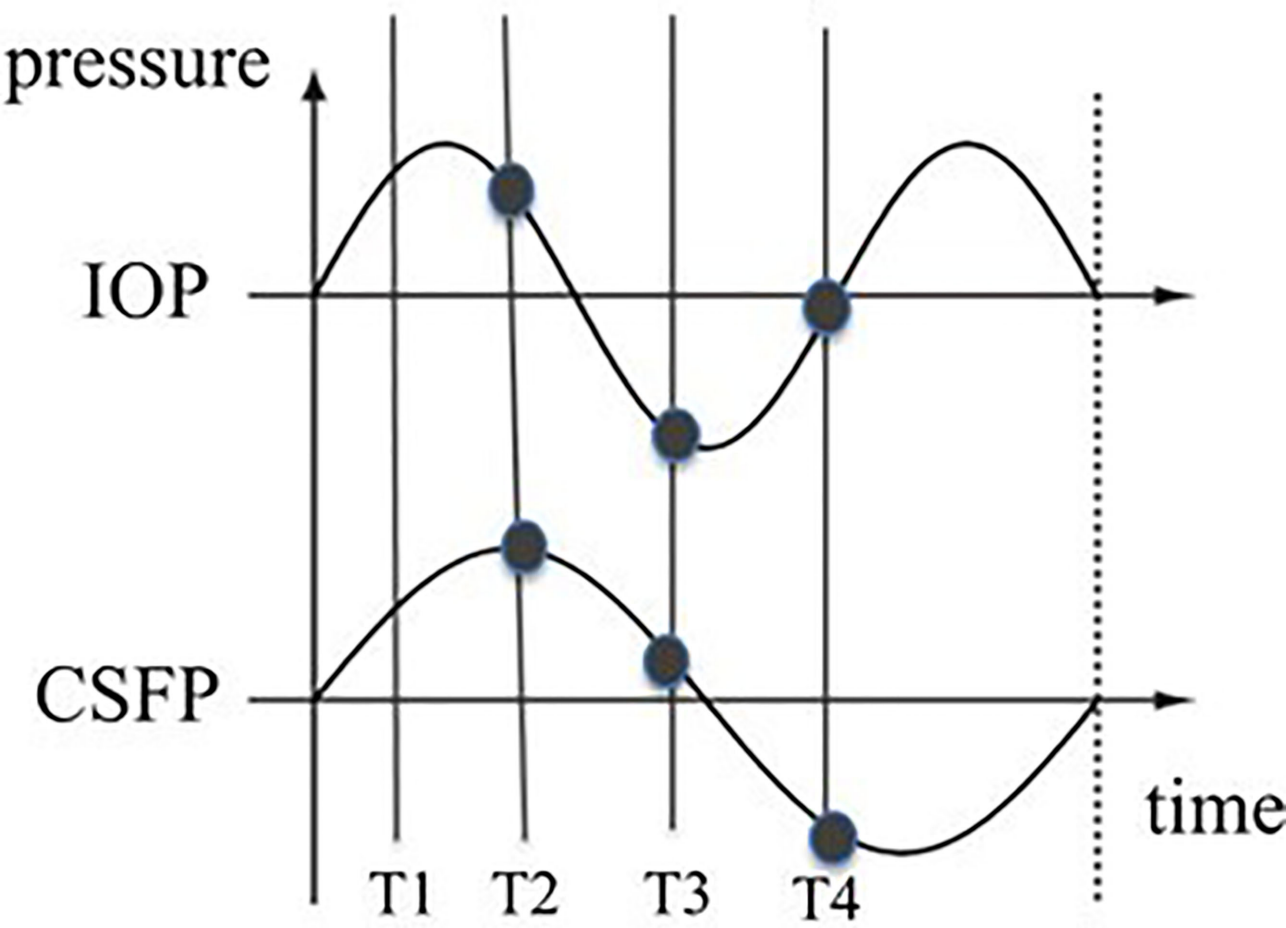
Possible wave forms of IOP and CSFP over time. The vertical lines represent the measuring times. The distance between the two curves at different times differ. The TLPG therefore varies over time.

**Figure 4:**
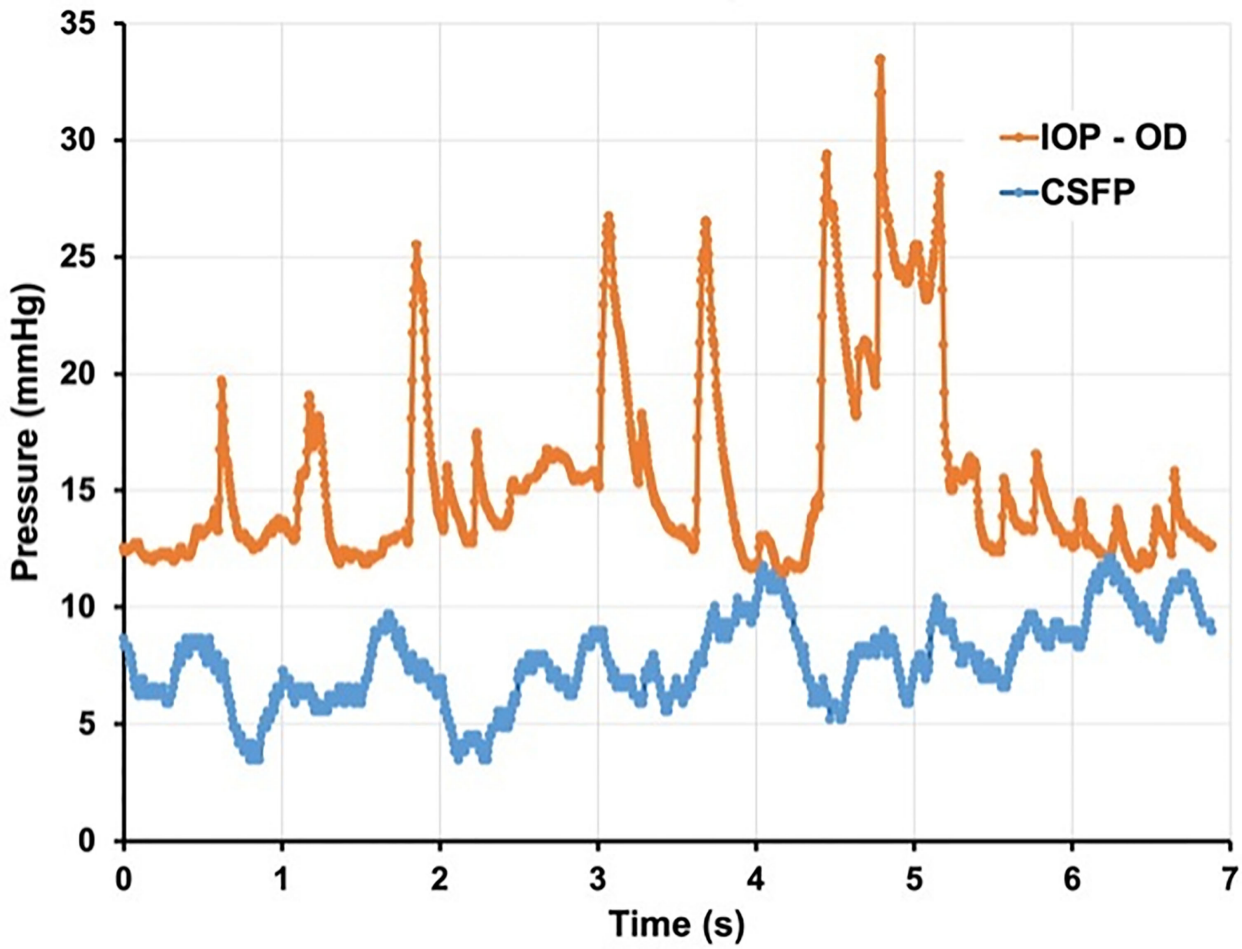
Seven seconds of continuous IOP and CSFP from non-human primate. demonstrating constant variation of TLPG and significant physiologic pressure variability. (Courtesy of Crawford Downs, PhD).

Glaucoma specialists are acutely aware of the limitations of a single snap-shot IOP reading in guiding treatment decisions as IOP fluctuates over time (37, 38). and is subject to normal physiologic diurnal variation (39, 40). Furthermore, several studies have identified exaggerated diurnal variation as an independent risk factor for progression (41). Much like IOP, CSFP fluctuates widely. A recent study by Downs et al. using implanted telemetry devices in nonhuman primates found that ICP was 92%–166% higher during sleeping hours than during waking hours (42). If we think about glaucomatous optic neuropathy as a product of the translaminar pressure, fluctuations in IOP and CSFP can be represented with a wave form. If IOP and CSFP are not measured simultaneously, then the alignment between these two forces becomes random. Thus, even in an ideal setting where both pressures are measured simultaneously the TLPG will constantly change unless the variations of IOP and CSFP are in perfect alignment – a dubious expectation (**Figures 3**, **4**).

Studies have validated the LP obtained in lateral decubitus for the measurement of ICP, but have also found that intracranial CSFP decreases to zero—or even subzero values—when standing (21). In general, CSFP is found to be in the low teens when supine and sub-atmospheric, and approaches equilibrium when sitting. As such, the TLPD is expected to be highest when upright. A study by Qvarlander et al. found a high degree of CSFP variation in the upright position compared with a low degree of variation in supine positioning, suggesting interindividual variation in capacity to regulate upright CSFP (43). These variations in tandem with the posture-dependence of CSFP further question the utility of a singular LP measurement without accounting for its diurnal and positional context.

## Estimation Equations

While IOP measurement is non-invasive and readily obtained in clinic, an LP is a sterile procedure of its own and is not concurrently attainable in the clinical setting. This makes clinical assessment of the TLPD technically challenging and highlights the importance of developing reliable non-invasive modalities for measuring CSF pressure. The Beijing Intracranial and Intraocular Pressure (iCOP) study developed a model for estimation of intracranial pressure by MRI-assisted orbital subarachnoid space measurement, and (44, 45) the study established a training group in order to validate its utility. However, a modification of the formula was used and termed *estimated cerebrospinal fluid pressure*. The following formula was used:

CSFP [mmHg] = 0.44 × Body Mass Index [kg/m(2)] + 0.16 × Diastolic Blood Pressure [mmHg]-0.18 × Age[Years]

No validation for this formula was ever given, and many inaccurately reference the Xie et al. manuscript as the derivation of this equation. What resulted was a series of studies that have been published using this unvalidated equation, contributing faulty data related to CSFP and ophthalmic disease research (46–57).

A subsequent study by Fleischman et al. identified limitations in multiple regression models to estimate CSFP without radiographic data (58). As expected, the estimation equation (and similarly-derived regression formulas from large datasets) poorly predicted CSFP in individuals. While we have identified and responded to studies that have used unvalidated estimation equations for CSFP-associated ophthalmic research, reviewers and journal editors should take note that these are not valid methods of conducting CSF-related research.

## Conclusion

IOP and CSFP are two highly dynamic pressures, which in clinical practice are assessed with snap-shot measurements of varying accuracy and applicability. The current methods used for the determination of the TLPD need to be improved in order to render more reliable data including modalities which allow for continuous measurement of IOP and CSFP. Similarly, appreciating the difference between orbital CSFP and lumbar CSFP is important and methods to easily and accurately report these fluid pressures need to be established. Discretization of the fluid pressure curves need to be performed in order to process the TLPD or gradient over time. As the role of the TLPD in the pathogenesis of glaucoma and other eye diseases continues to be explored, the nuances of CSF behavior and the technical limitations in measuring intra-orbital CSFP must be appreciated.

## Author Contributions

All authors listed have made a substantial, direct, and intellectual contribution to the work, and approved it for publication.

## Conflict of Interest

The authors declare that the research was conducted in the absence of any commercial or financial relationships that could be construed as a potential conflict of interest.

## Publisher’s Note

All claims expressed in this article are solely those of the authors and do not necessarily represent those of their affiliated organizations, or those of the publisher, the editors and the reviewers. Any product that may be evaluated in this article, or claim that may be made by its manufacturer, is not guaranteed or endorsed by the publisher.
